# The *Drosophila* DEG/ENaC PPK12 is a Na^+^ leak channel with a low Na^+^ affinity

**DOI:** 10.1038/s41598-025-10609-7

**Published:** 2025-07-28

**Authors:** Lu Qin, Nikita Komarov, Cornelia Fritsch, Simon G. Sprecher, Stefan Gründer, Dominik Wiemuth

**Affiliations:** 1 Institute of Physiology, RWTH Aachen University, Pauwelsstrasse 30, 52074 Aachen, Germany; 2 Department of Biology, University of Fribourg, Chemin du Musée 10, Fribourg, CH-1700 Switzerland

**Keywords:** Ion channel, Larvae, Salt sensation, Taste, Ion channels in the nervous system, Molecular evolution, Permeation and transport

## Abstract

DEG/ENaC ion channels have various functions in different organisms. In *Drosophila*, DEG/ENaCs are named Pickpockets (PPKs) and form a large insect-specific radiation with seven subfamilies containing 31 members. Several different functions have been proposed for PPKs, including salt and water taste. However, despite their many functions, most PPKs have not been functionally characterized in heterologous expression systems, leaving their functional properties unknown. Here, we expressed six PPKs in *Xenopus* oocytes, which are expressed in the chemosensory system of *Drosophila* larvae. We found that PPK12 forms a constitutively open ion channel that is permeable to Na^+^ ions. PPK12 currents do not saturate even at high Na^+^ concentrations, suggesting that PPK12 may be involved in sensing high salt concentrations. Our study shows that at least some PPKs are amenable to functional characterization in *Xenopus* oocytes, allowing to elucidate the relation of their functional properties with their proposed functions in the organism.

## Introduction

The degenerin/epithelial Na^+^ channel (DEG/ENaC) family evolved early in metazoans^1,2^ and family members have strikingly diverse functional properties in different animals: some are mechanosensitive like degenerins^3^some are constitutively open like ENaC^4^and others are opened by peptides^5,6^ or protons^7^; the functional properties of many other DEG/ENaCs are unknown. Correspondingly, DEG/ENaCs serve many different functions, including mechanosensation, proprioception, Na^+^ reabsorption, synaptic transmission, and pH sensing. The range of proposed functions suggests that these receptors may be rapidly evolving and diversifying. Despite their variable functions, members of the DEG/ENaC family share a conserved structure; they are trimers, where each subunit has a conserved topology with two transmembrane domains (TMDs) and a large extracellular domain (ECD), which may contain a ligand-binding region^8^. Typically, DEG/ENaCs are Na^+^ channels, but DEG/ENaCs that are unselective for cations with a high Ca^2+^ permeability are also known^9^. The prototypical blocker of DEG/ENaCs is amiloride^10^; IC_50_ values of DEG/ENaCs for amiloride range from 0.1 µM for ENaC^11^, ~ 10 µM for proton-sensitive ASICs^7^, to ~ 100 µM for peptide-gated HyNaCs^12^. Compared with amiloride, the diarylamidine diminazene, another pore blocker of some DEG/ENaCs^13^has an approximately tenfold higher affinity for ASICs^14^ and > 100-fold higher affinity for HyNaCs^15^.

In insects, many ion channel families critical to sensory neuron function have been identified, including ionotropic receptors (IRs) and odorant receptors (ORs), which constitute insect-specific groups^16–18^. Similarly, insects possess DEG/ENaCs, named Pickpockets (PPKs), which form a large insect-specific radiation, suggesting that PPKs expanded from a common precursor into a large variety of ion channels that form seven subfamilies^19,20^. *Drosophila melanogaster* has 31 PPK genes^19^for which many different functions have been proposed, including liquid clearing in the tracheal system^21^, salt taste^22^, detection of high salt concentrations^23^, gustatory water reception^24,25^, pheromone sensing^26^, control of locomotion^27–29^, and mechanical nociception^29–31^. In some cases, functional studies of PPK-expressing neurons have found that secondary genes are required for proper function in salt detection via Serrano (sano)^23^ and mechanical nociception via Piezo^32^, suggesting a more complex interaction between PPK protein family members and modulatory proteins. Interestingly, in some cases, PPKs may themselves be modulators, such as in some olfactory neurons where one PPK family member was found to modulate calcium signal amplification^33^. Furthermore, non-neuronal expression of some PPKs has also been noted, indicating a broader role in cell function within *Drosophila*, such as cell osmolarity regulation. However, the functions and activating stimuli of many PPKs remain unknown. In addition, only a few PPKs have been functionally characterized in heterologous expression systems^34,35^such that their functional properties remain unknown.

In this study, we screened six PPKs from four different subfamilies that have been reported to be expressed in the chemosensory system^36,37^. Furthermore, the receptors were selected due to their proposed role in chemosensation, as the neurons expressing them have been implicated in food-related behaviours^22,33^. This makes these receptors a point of interest, as they may provide a direct link between environmental sensing and behavioural output. Among these, we found that PPK12 is a constitutively open ion channel that is weakly selective for Na^+^ with a low affinity for Na^+^. We speculate that PPK12 may be involved in the detection of high Na^+^ concentrations.

## Results

**Fig. 1:**
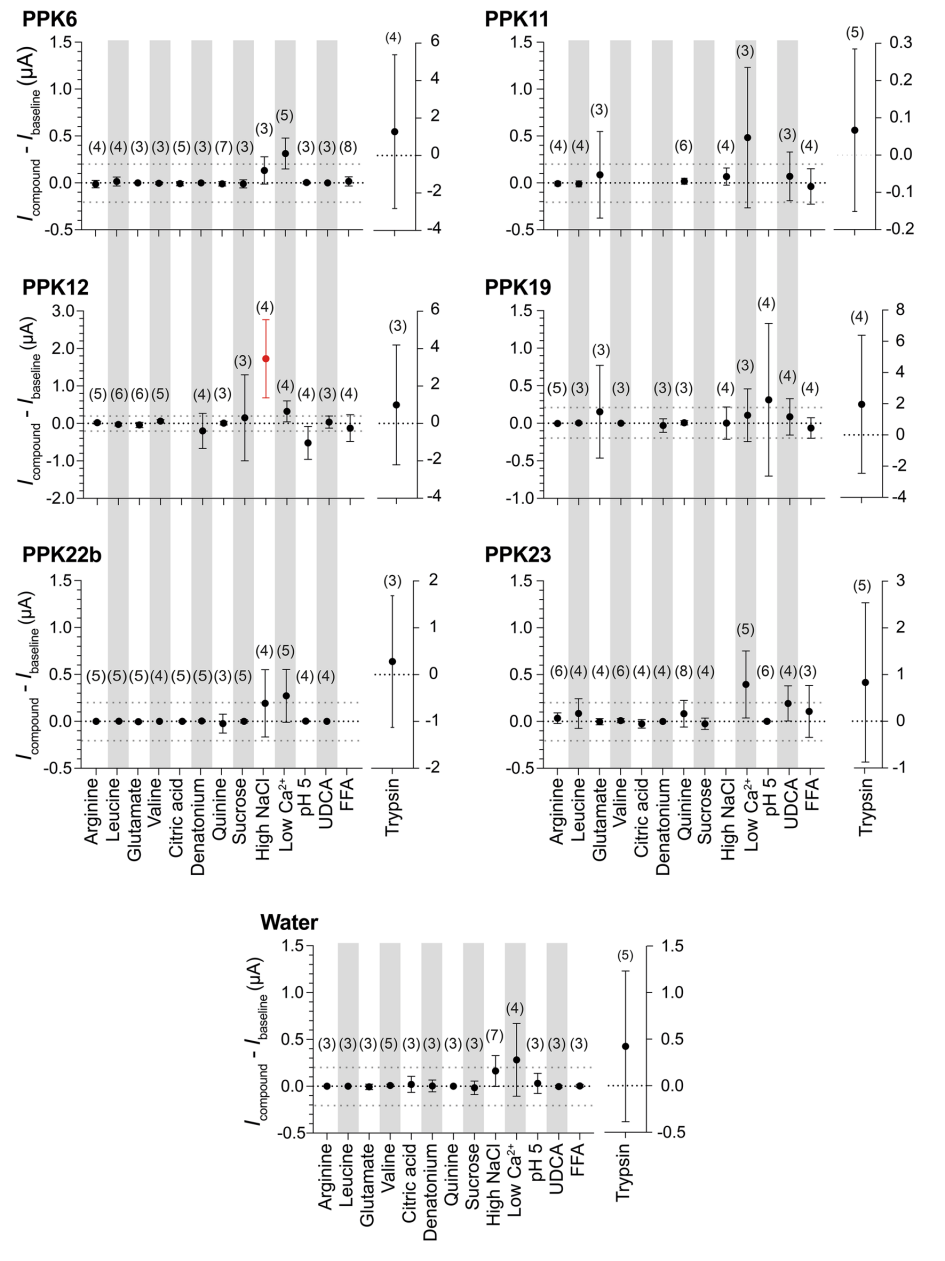
Summary of the effects of different substances on six different PPKs. Substances indicated at the bottom were used at concentrations of 10 mM (arginine, leucine, glutamate, valine, and citric acid), 1 mM (quinine, denatonium, and FFA), 100 mM (sucrose), 300 mM (high salt), 10 nM (low Ca^2+^), or 3 mM (UDCA). Dots represent the mean and error bars the 95% confidence interval (CI). We considered responses > 0.2 µA as meaningful (dashed lines). Note that for trypsin (2 mg/ml), the variability was large, and results are shown on a different scale. Only for high NaCl and PPK12, the 95%-CI was > 0.2 µA. The number n of independent oocytes is indicated above dots. When n was < 3, the results are not shown. Results for water-injected oocytes are shown at the bottom. UDCA, ursodeoxycholic acid; FFA, flufenamic acid.

To characterize the response of PPK channels to different stimuli, we initially co-expressed six PPK channels in *Xenopus* oocytes and superfused oocytes with various taste substances (amino acids, bitter compounds, salts, and acids) and bile acids, which activate the mammalian bile acid-sensitive ion channel (BASIC)^38^. Moreover, we reduced [Ca^2+^]_e_ to approximately 10 nM, which opens some DEG/ENaCs^15,39^, and treated oocytes with trypsin, which activates ENaCs^40^. Neither of these substances induced currents in oocytes co-expressing the six PPKs or water-injected control oocytes. We then expressed the six PPK channels individually in oocytes. The substances did not affect PPK activity, with the exception of PPK12-expressing oocytes, where high salt concentration (300 mM NaCl) increased currents (Fig. 1). Therefore, we analysed PPK12 further. While our preliminary first analysis did not yield evidence for activity in oocytes of the other five PPKs, a more in-depth analysis would be necessary to characterize their expression and properties in oocytes.

**Fig. 2:**
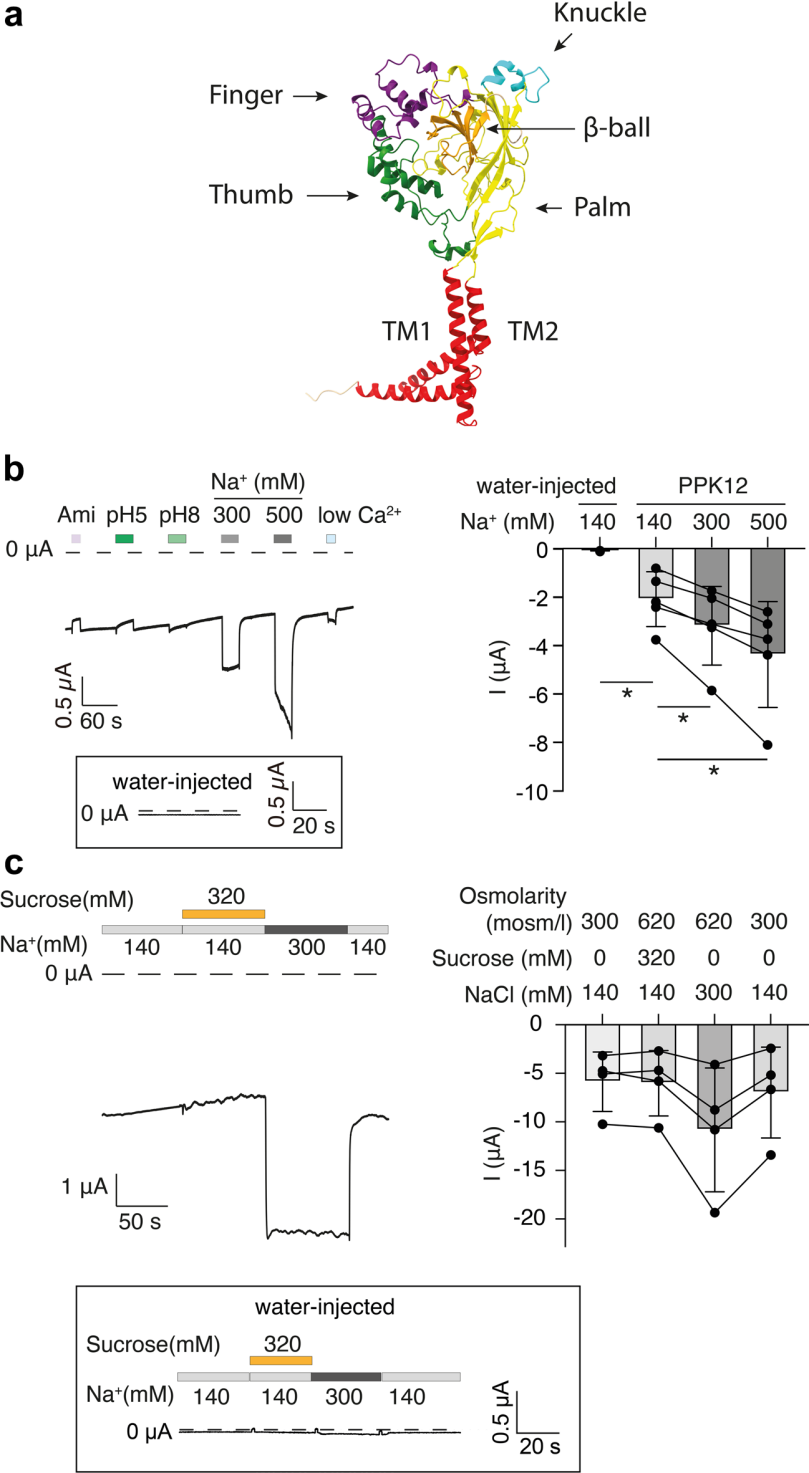
PPK12 mediates constitutive Na^+^ currents in *Xenopus* oocytes. (**a**) Alphafold structure of PPK12. The model is available at https://alphafold.ebi.ac.uk/entry/Q9W250. Different domains are indicated. (**b**) Left, representative current trace of an oocyte expressing PPK12. The oocyte was superfused with solutions containing either 100 µM amiloride (Ami), pH 5.0, pH 8.0, 300 mM NaCl, 500 mM NaCl, or low Ca²⁺ (approximately 10 nM; see Methods). The inset shows a representative trace of a water-injected oocyte; the dashed line represents the 0 current level. Right, current amplitude of PPK12-expressing oocytes at different Na^+^ concentrations (mean ± SEM). *n* = 5. (**c**) Left, representative current trace of a PPK12-expressing oocyte superfused with solutions of different osmolarities or NaCl concentrations. Right, summary of current amplitudes (mean ± SEM). The osmolarity was either 300 or 620 mosm/l. *n* = 5. The inset shows a current trace of a water-injected oocyte, which is representative for five similar measurements; the dashed line represents the 0 current level. *, *P* < 0.05.

Prediction of the PPK12 structure using AlphaFold suggests that a single PPK12 subunit shares the main domains–two TMDs, palm, thumb, finger, knuckle and β-ball–with chicken ASIC1 ^8^ (Fig. 2a). However, there are some notable differences, for example α2 of the finger, which is at the top of the ECD in cASIC1, is tilted towards the thumb domain in the predicted structure of PPK12. Expression of PPK12 in *Xenopus* oocytes induced large constitutive currents (3.2 ± 0.6 µA) compared to water-injected control oocytes (0.1 ± 0.02 µA; *P* = 0.035; Fig. 2b). The constitutive currents were slightly decreased by acidic pH (pH 5.0) and increased by alkaline pH (pH 8.0). In addition, the common DEG/ENaC blocker amiloride (100 µM) weakly inhibited the current, whereas the removal of extracellular Ca^2+^ increased it. Moreover, the constitutive current strongly increased in the presence of higher NaCl concentrations (300 mM, *P* = 0.030, and 500 mM, *P* = 0.029) (Fig. 2b). To test whether the larger current amplitude with high NaCl concentrations was due to the higher osmolarity or to the higher Na^+^ concentration, we applied an isosmotic control solution (~ 300 mosm/l, 140 mM Na^+^) and hyperosmotic solutions (~ 620 mosm/l) containing either 140 mM or 300 mM Na^+^ to PPK12-expressing oocytes. When the osmolarity was increased from ~ 300 to ~ 620 mosm/l at a constant Na^+^ concentration (140 mM), the constitutive currents did not increase. However, when the Na^+^ concentration increased from 140 mM to 300 mM at the same osmolarity (~ 620 mosm/l), the constitutive current rapidly and strongly increased (*P* = 0.051; Fig. 2c). Importantly, in water-injected oocytes, 300 mM Na^+^ did not induce an increase in the inward current (Fig. 2c). Taken together, these findings suggest that PPK12 is a constitutively open ion channel that conducts Na^+^ ions.

**Fig. 3:**
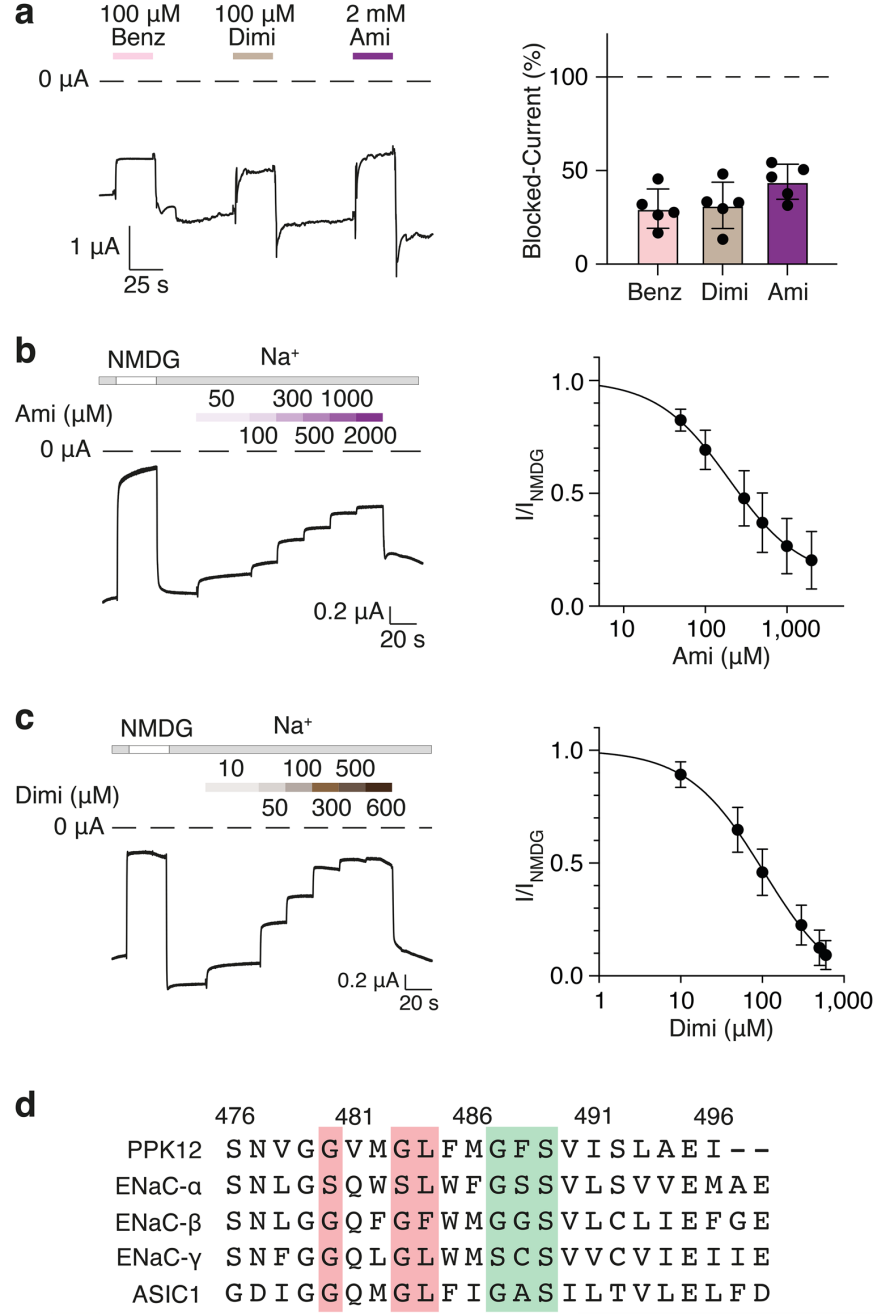
PPK12 is blocked by diminazene and amiloride. (**a**) Left, representative current trace showing inhibition of the constitutive current of PPK12-expressing oocytes by 100 µM benzamil (Benz), 100 µM diminazene (Dimi), or 2 mM amiloride (Ami). Right, quantification (mean ± SEM). *n* = 5. Note that only part of the constitutive current was carried by PPK12, which underestimates the block of PPK12 in this experiment, and that the brief outward tail currents are probably unspecific artifacts associated with solution exchange. (**b**) Left, representative current trace recorded in the presence of increasing concentrations of amiloride. Right, concentration-dependent inhibition of PPK12. Currents were normalized to the current that was reduced by a Na^+^-free solution (NMDG), *n* = 10. (**c**) As in b) but for diminazene. *n* = 8. (**d**) Sequence alignment of TMD2, containing the pore region, of PPK12, rat αENaC, βENaC, γENaC, and rat ASIC1a. Amino acids that are important for amiloride and diminazene block of ENaC and ASIC1a are shown on red background and amino acids of the selectivity filter on green background.

Three typical DEG/ENaC blockers–amiloride, its analog benzamil, and diminazene–partially and reversibly inhibited constitutive currents in PPK12-expressing oocytes at a concentration of 100 µM (benzamil, diminazene) or 2 mM (amiloride) (Fig. 3a). We determined the apparent affinities of PPK12 for amiloride and diminazene. To better estimate the contribution of PPK12 to the constitutive current, we first applied a solution in which Na^+^ was replaced with the large cation NMDG^+^. The large inward current of PPK12-expressing oocytes was strongly diminished (Fig. 3b), suggesting that NMDG cannot permeate PPK12 and that PPK12 mediates a large proportion of the constitutive currents in PPK12-expressing oocytes. The NMDG-sensitive current was used as a proxy for the PPK12-mediated current. The constitutive inward current gradually decreased with increasing concentrations of both inhibitors (Fig. 3b and c). PPK12 was almost completely blocked by 600 µM diminazene, whereas it was only partially blocked even by 2 mM amiloride. The apparent affinity of PPK12 for diminazene (IC_50_ = 110 ± 10 µM; Hill-coefficient H = 0.96 ± 0.04) was ~ two-fold higher than that for amiloride (IC_50_ = 220 ± 20 µM; H = 0.99 ± 0.03). Together, these results show that PPK12 has pharmacological characteristics similar to those of other DEG/ENaCs, but that its affinity for the pore blockers amiloride and diminazene is rather low. Amiloride and diminazene block ENaCs and ASIC1a by binding to the outer pore region^14,41^. One amino acid in this region is an Gln in βENaC, γENaC, and ASIC1a^14,41^ but a Val in PPK12 (V481; Fig. 3d), which may explain the relatively low amiloride and diminazene affinity of PPK12.

**Fig. 4:**
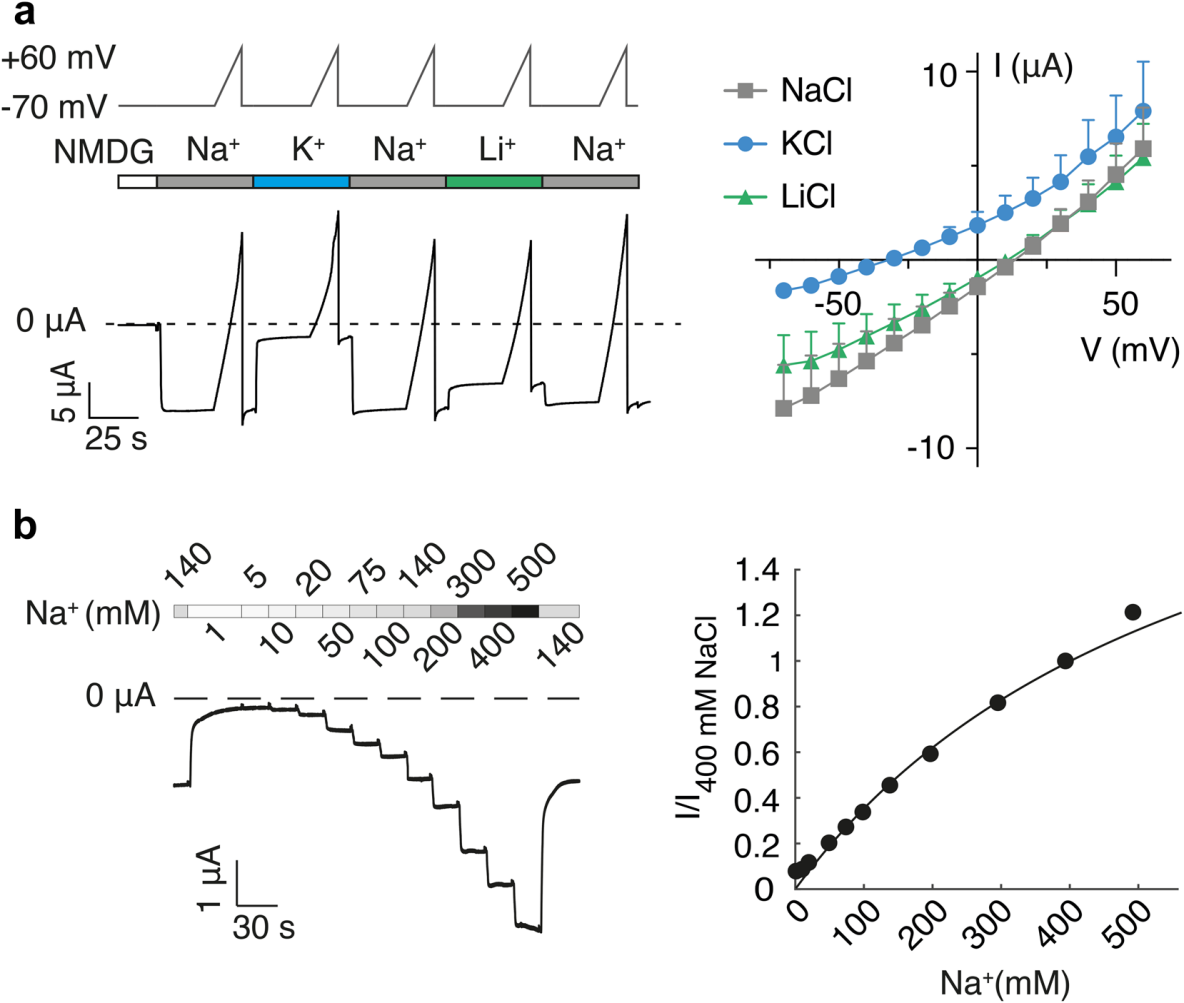
PPK12 is slightly Na^+^-selective and has low Na^+^ affinity. (**a**) Left, representative current trace (bottom) from a PPK12-expressing oocyte. The main extracellular cation was exchanged (140 mM NaCl, KCl, or LiCl), and the membrane voltage was changed from a holding potential of −70 mV to + 60 mV in 15 s, using a ramp protocol (see scheme at the top). Right, mean current-voltage relationships. For Na^+^, the mean current-voltage relationship from the first ramp is shown. Dots represent the mean and bars the SEM, *n* = 6. (**b**) Left, representative current trace of a PPK12-expressing oocyte illustrating the dependence of the current amplitude on the Na^+^-concentration. Right, mean concentration-response curve for Na^+^. The curve represents a fit to the Michaelis-Menten equation. Dots represent the mean and bars the SEM, *n* = 8.

Next, we studied the ion selectivity of PPK12. DEG/ENaCs typically conduct Na^+^ and exhibit a relatively high selectivity for Na^+^ over K^+^. Oocytes superfused with extracellular solutions containing 140 mM NaCl, 140 mM LiCl, or 140 mM KCl (Fig. 4a) had reversal potentials E_rev_ of 19 ± 5, 16 ± 5, and − 22 ± 7 mV, respectively. Using the Goldman-Hodgkin-Katz equation, we calculated permeability ratios P_Na_/P_Li_ of 1.2 and P_Na_/P_K_ of 6.1. Although these values were obtained for the constitutive leak current and are therefore only approximations, they indicate that PPK12 is a cation channel with a slight selectivity for Na^+^ and a permeability sequence of Na^+^ ≥ Li^+^ > K^+^. The ion pore of DEG/ENaCs is mainly lined by TMD2 residues with the strongest constriction in the middle of TMD2, formed by a GXS motif^42^. TMD2 of PPK12 is overall well conserved with that of ASICs and ENaC (Fig. 3d), supporting conserved pore properties and Na^+^ selectivity.

To analyse the affinity of PPK12 for Na⁺, we measured the concentration dependence of the inward currents with Na⁺ concentrations ranging from 1 to 500 mM. The non-conducting ion NMDG was used to replace Na^+^ and maintain identical osmolarity for each Na⁺ concentration. The constitutive inward currents of PPK12-expressing oocytes increased with increasing Na^+^ concentration and did not saturate at 500 mM (Figs. 2b and 4b), which explains the increased current amplitudes initially observed at higher NaCl concentrations (Fig. 2b). Using the Michaelis-Menten equation, the mean Km value was estimated to be ~ 630 mM.

## Discussion

In this study, we found that PPK12 can be expressed in *Xenopus* oocytes, where it forms constitutively open Na^+^ channels that are inhibited by amiloride and diminazene with low affinity. Expression of PPK12 has so far only been experimentally validated in a single gustatory neuron in the *Drosophila* larval primary taste center, the Terminal Organ (TO)^37^, suggesting a role in sensory function. ENaC, which is involved in Na^+^ reabsorption in different epithelia, has a Na^+^ affinity of ~ 5 mM^11^, thus ~ 100-fold higher than that of PPK12. The low Na^+^ affinity of PPK12 renders it particularly suitable for sensing high Na^+^ concentrations. For instance, a population existing in marine-adjacent or desiccating environments may need to avoid excessive salt levels that could contaminate their food sources. On the other hand, the typical diet of *Drosophila* consists of decaying fruit that have low salt levels, which may instead require to seek additional Na^+^ sources to maintain ionic homeostasis. The full extent of the capabilities of *Drosophila* salt sensing is not understood, however several mechanisms have been elucidated, suggesting that these animals are able to discriminate salt levels in a variety of physiological conditions^23,43,44^. In addition to expression in a sensory neuron, transcriptomic analysis of the adult *Drosophila* suggests a low expression level of PPK12 across a range of tissues^45^, potentially suggesting a role in osmotic homeostasis in a variety of cell types. In summary, we speculate that PPK12 may be involved in sensing and perhaps also reabsorption of Na^+^.

PPK12 belongs to PPK subfamily V and is closely related to PPK5 and PPK8 ^19,20^, the functions of which are unknown, but which may have functional properties similar to PPK12. The Na^+^ affinities of PPK5 and PPK8 are unknown and may be lower than that of PPK12. The next most closely related PPK in subfamily V is PPK28. Interestingly, PPK28 has been proposed to be an osmosensitive ion channel that responds to hypo-osmolarity and mediates water sensitivity in *Drosophila*^24,25^. This implies that the channel is closed under normal and under high-salt conditions but opened under low-salt conditions–very different from PPK12. In oocytes, PPK12 did not react to changes in osmolarity for one minute (Fig. 2c), suggesting that it is not sensitive to osmolarity. In future studies, it will be interesting to compare the gating mechanisms of the close relatives PPK28 and PPK12.

PPK1, PPK2, and PPK26 are further members of subfamily V, which are more distantly related to the PPK12/PPK5/PPK8/PPK28 group^19,20^. PPK1 and PPK2 (also named Ripped Pocket, RPK) are the founding members of the PPK family^34,35,46^. PPK2 has been successfully expressed in *Xenopus* oocytes, where it induces small constitutive currents (on the order of 0.1 µA)^34,35^. It contributes to the sense of touch in *Drosophila* larvae^47^. In contrast, PPK1 does not induce currents in oocytes^34,46^. Together with PPK26 it is involved in regulating locomotion and mechanical nociception in *Drosophila* larvae^27,29–31^suggesting that PPK2, PPK1, and PPK26 are mechanosensitive ion channels. The close evolutionary relationship between the salt-/osmosensing PPK12/PPK5/PPK8/PPK28 group and the mechanosensing PPK1/PPK2/PPK26 group in subfamily V is reminiscent of the relatively close relationship between salt-transporting ENaCs and mechanosensitive degenerins within clade B of DEG/ENaCs^1,2^.

Our study has a few limitations. First, as several PPKs are expressed in the chemosensory system of *Drosophila* larvae, it is possible that PPK12 forms heteromeric channels with other PPKs, similar to ENaC^11^ and other DEG/ENaCs^15,48^. This could result in higher Na^+^ affinity or other distinct properties. While our study did not reveal an indication for heteromers of PPK12 with PPK6, PPK11, PPK19, PPK22b, or PPK23, we cannot exclude the formation of PPK12 heteromers with these or other PPKs. Second, PPK12 may be modulated by other proteins, like sano, which could change its properties. And third, while expression in *Xenopus* oocytes usually allows the faithful characterization of the basic biophysical properties of ion channels, it is possible that PPK12 may have distinct properties in its native cellular environment.

Also for some of the remaining 24 *Drosophila* PPKs of other subfamilies, functions have been proposed, which range from liquid clearing in the tracheal system^21^salt taste^22^pheromone sensing^26^to detection of high salt concentrations^23^. Thus, PPKs appear to be versatile channels that have been co-opted for a variety of physiological functions in insects. The evidence for their role in these functions is usually based on genetic studies with loss-of-function alleles of individual channels. To our knowledge, except for PPK2, the functional properties of PPKs have not been characterized in heterologous systems. Our study demonstrates that at least some PPKs are amenable to functional characterization in *Xenopus* oocytes, allowing to elucidate, in the future, the molecular properties of individual PPKs that endow them with the capacity to serve diverse functions in *Drosophila* and other insects.

## Methods

### Cloning of PPKs

The coding sequences of *ppk6*, *ppk11*, *ppk12*, *ppk19*, *ppk22 RB*, and *ppk23 RD* were synthetized and inserted into pBlueScript II SK(+) (Stratagene) at BioCat (Heidelberg, Germany). The different *ppk* coding sequences were then PCR-amplified to add a vertebrate Kozak sequence (CCACC) and appropriate restriction sites (Table 1) for cloning into the *Xenopus* expression vector pRSSP6009 ^49^. After cloning, the sequence of the plasmids was verified with primer “RSSP seq re” binding to the *Xenopus* globin 3’UTR of the vector to exclude the presence of PCR errors.

**Table 1:** Primers used for cloning and sequencing of PPK constructs in 5’- to 3’- orientation. Coding sequences in capital letters; attached sequences in small letters; restriction sites in italic; Kozak sequences in bold.

Primer	sequence
ppk6 Start *Xho* fw	ca*ctcgag***ccacc**ATGATAGAATCGGGAAAGTGGCCAAC
ppk6 end *Kpn* re	ta*ggtacc*TTATTGCCATGCCTTTCTGGGTTGC
ppk11 Start *Xho* fw	tt*ctcgag***ccacc**ATGTCCGACGTTCCAGGAGAG
ppk11 end Kpn re	ac*ggtaCC*TAATTAGCCGGCCCTAATGACC
ppk12 Start *Xho* fw	ta*ctcgag***ccacc**ATGGAACCGTCGCCGTC
ppk12 end *Kpn* re	gt*ggtacc*TTATTGGTAATTTAATCCTTTCGTACTTTTAGCATTTTCGTAGG
ppk19 Start *Xho* fw	ac*ctcgag***ccacc**ATGTTGCTGTACACCAAGGAACTTG
ppk19 end *Kpn* re	ta*ggtacc*GCTACTTAGTATACTCTTTCAATTTTATGCGCGC
ppk22 Start *BglII* fw	aa*agatct*g**ccacc**ATGGTCAAGCTGGCATCAACATCC
ppk22 end *Age* re	ac*accggT*CAAGGACATATGTACAACTTCTTCGTTTCC
ppk23 Start *Pst* fw	tc*ctgcag***ccacc**ATGCCGCAAGAGAAACGCC
ppk23 CD end *Kpn* re	ct*ggtacc*GCTTCAGGGCATGTACTGAGAATGATC
RSSP seq re	GCTTAGAGACTCCATTCG

### Electrophysiology

Stage V–VI oocytes were surgically removed under anaesthesia (2.5 g/L tricaine methanesulfonate for 20 min) from adult *Xenopus laevis* females. Anesthetized frogs were euthanized by decapitation following the final oocyte harvest. Animal care and experiments were conducted under protocols approved by the State office for nature, environment and consumer protection (LANUV) of the State North Rhine-Westphalia (NRW) and were performed in accordance with LANUV NRW guidelines (approval # 81-02.04.2019.A356). All procedures complied with the ARRIVE guidelines.

Before injection, oocytes were incubated with collagenase (1.67 mg/ml) in OR2 (in mM: 82.5 NaCl, 2.5 KCl, 1 Na_2_HPO_4_, 1 MgCl_2_, 1 CaCl_2_, 5 HEPES, 0.5 g/L PVP, pH 7.3) for two hours. Oocytes were injected with 10–15 ng cRNA and incubated in OR2 at 19 °C for 24–72 h. For oocytes injected with ppk12, we used an OR2 in which we replaced most Na^+^ by NMDG^+^ (OR2-NMDG; in mM: 5 NaCl, 77.5 NMDG, 2.5 KCl, 1 Na_2_HPO_4_, 1 CaCl_2_, 1 MgCl_2_, 5 HEPES, 0.5 g/L PVP, pH 7.3)^49^.

Whole-cell currents were recorded with a TurboTec 03X amplifier (npi electronic, Tamm, Germany) using an automated, pump-driven solution exchange system, together with the oocyte-testing carrousel controlled by the interface OTC-20 (npi electronic). We used glass capillaries from Science Products (GB150TF-10; Hofheim, Germany). Data acquisition and solution exchange were managed using CellWorks version 6.2.2 (npi electronic). Unless otherwise noted, data were obtained at a holding potential of −70 mV. Data were filtered at 20 Hz and acquired at 1 kHz. All experiments were performed at room temperature (20–25 °C). The standard bath solution contained 140 mM NaCl, 10 mM HEPES, 1.8 mM CaCl_2_, and 1 mM MgCl_2_ at a pH of 7.4. Low Ca^2+^ bath solution contained 140 mM NaCl, 10 mM HEPES, 10 nM CaCl_2_ (instead of 1.8 mM CaCl_2_), but we did not buffer the Ca^2+^ concentration to a precise concentration. For bath solutions with acidic or basic pH, the pH was adjusted to the desired value using HCl or NaOH. In some experiments, 140 mM NaCl in the bath solution was replaced with 140 mM LiCl, KCl, or NMDG. In some bath solutions, the osmolarity was adjusted using 320 mM sucrose. The osmolarity indicated in the text and figures is an approximation and was calculated assuming complete dissociation of salts.

### Data analysis

Data were collected from oocytes of at least two different animals. Data were analysed using the software IgorPro version 8.04 (WaveMetrics, Portland, USA) and Prism 9 (GraphPad, Boston, USA). Results are reported as the mean ± S.E.M., except for Fig. 1, which shows the mean and 95% confidence intervals. Statistical significance was assessed using one-way ANOVA followed by Dunnett´s post-test (Fig. 2b) or paired Student’s t-test (Fig. 2c); the data was normally distributed (Shapiro-Wilk test).

Concentration - response curves were fitted by a Hill function (Eq. 1)

1 $$\:{I}_{x}=\frac{1}{1+{\left(\frac{\left[x\right]}{IC50}\right)}^{H}}$$.

where *I*_x_ is the current at a given inhibitor concentration, [*x*] is the concentration of the inhibitor, *IC*_50_ is the concentration at which 50% of the current was inhibited, and *H* represents the Hill coefficient.

To calculate the affinity for Na^+^, current amplitudes at different Na^+^ concentrations were fitted with the Michaelis-Menten function (Eq. 2)

2 $$\:I\:=\:\frac{{I}_{max}*\left[c\right]}{{K}_{m}+\left[c\right]}$$.

where *I*_max_ is the maximum current, [*c*] is the Na^+^ concentration, and *K*_m_ is the Na^+^ concentration at which half-maximum saturation occurred.

To determine ion selectivity, reversal potentials were determined in a bath solution containing 140 mM Na^+^, K^+^, or Li^+^ using a voltage ramp protocol (from − 70 to + 60 mV in 15 s). The permeability ratios, P_Na_/*P*_X_, were calculated with the Goldman-Hodgkin-Katz equation (Eq. 2), using the measured reversal potentials.

2 $$\:\:\frac{{P}_{{Na}^{+}}}{{P}_{{X}^{+}}}=\:{e}^{\frac{F\:({E}_{{rev-Na}^{+}}\:-\:{E}_{{rev-X}^{+}})}{RT}\:}$$.

where *F* is the Faraday constant, *R* is the gas constant, *T* is the absolute temperature (294 K), and *E*_rev_ is the reversal potential. For Na^+^, we ran three ramps to evaluate a possible shift of the reversal potential (see Fig. 4a). The reversal potentials E_rev, Na+_ determined using the three ramps were 19.4 ± 5.1 mV, 19.5 ± 5.0 mV, and 16.3 ± 5.6 mV, respectively (*n* = 6). While the first two reversal potentials were not different from each other (*P* = 0.91), the third reversal potential was slightly shifted (*P* = 0.027), probably due to an increased intracellular Na^+^ concentration. Therefore, to calculate permeability ratios, we used the Na^+^ reversal potential measured using the ramp preceding the respective ion with which it was compared (first ramp for comparison with K^+^ and second ramp for comparison with Li^+^).

## Data Availability

All data generated or analysed during this study are included in this manuscript. The PPK12 structure from Alphafold is available at https://alphafold.ebi.ac.uk/entry/Q9W250.
